# Improving emergency obstetric referral systems in low and middle income countries: a qualitative study in a tertiary health facility in Ghana

**DOI:** 10.1186/s12913-020-4886-3

**Published:** 2020-01-10

**Authors:** Anita Anima Daniels, Aaron Abuosi

**Affiliations:** Department of Public Administration and Health Services Management, University of Ghana Business School, University of Ghana, Legon, Ghana

**Keywords:** Maternal health, Emergency obstetric care, Referral system, Tertiary health facility, Ghana

## Abstract

**Background:**

Timely access to emergency obstetric care is crucial in preventing mortalities associated with pregnancy and childbirth. The referral of patients from lower levels of care to higher levels has been identified as an integral component of the health care delivery system in Ghana. To this effect, in 2012, the National Referral Policy and Guidelines was developed by the Ministry of Health (MOH) to help improve standard procedures and reduce delays which affect access to emergency care. Nonetheless, ensuring timely access to care during referral of obstetric emergencies has been problematic. The study aimed to identify barriers associated with the referral of emergency obstetric cases to the leading national referral centre. It specifically examines the lived experiences of patients, healthcare providers and relatives of patients on the referral system.

**Methods:**

Korle Bu Teaching Hospital, Accra was used as a case study in 2016.The qualitative method was used and in-depth interviews were conducted with 89 respondents: healthcare providers [*n* = 34];patients [*n* = 31] and relatives of patients [*n* = 24] using semi-structured interview guides. Purposive sampling techniques were used in selecting healthcare providers and patients and convenience sampling techniques were used in selecting relatives of patients.

**Results:**

The study identified a range of barriers encountered in the referral process and broadly fall under the major themes: referral transportation system, referrer-receiver communication barriers, inadequate infrastructure and supplies and insufficient health personnel. Some highlights of the problem included inadequate use of ambulance services, poor management of patients during transit, lack of professional escort, unannounced emergency referrals, lack of adequate information and feedback and limited supply of beds, drugs and blood. These findings have implications on type II and III of the three delays model.

**Conclusions:**

Initiatives to improve the transportation system for the referral of obstetric emergencies are vital in ensuring patients’ safety during transfer. Communication between referring and receiving facilities should be enhanced. A strong collaboration is needed between teaching hospitals and other stakeholders in the referral chain to foster good referral practices and healthcare delivery. Concurrently, supply side barriers at referred facilities including ensuring sufficient provision for bed, blood, drugs, and personnel must be addressed.

## Background

Global attention given to maternal health has been geared towards reducing maternal mortality which has over the years showed great discrepancy between Low, Middle and High Income Countries. The World Health Organisation reports that about 800 women die from pregnancy- or childbirth-related complications around the world every day [1]. However, 99% of all maternal deaths occur in Low and Middle Income Countries (LMICs). Reducing maternal mortality has been rolled on into the Sustainable Development Goal (SDG) 3 which seeks to ensure healthy lives and promote well-being for all at all ages. This goal targets to reduce the global maternal mortality ratio to less than 70 per 100,000 live births by the year 2030. Access to appropriate health care including skilled birth attendance at delivery and timely referrals to access emergency obstetric care services can greatly reduce maternal deaths and disabilities [2, 3]. Timely access to emergency obstetric care is seen as crucial to avert any negative outcome that could result from unpredictable complications that could arise during pregnancy and childbirth. Timely access to emergency care is thus seen as an indication of a successful health system. The inability of most women to access timely Emergency Obstetric Care (EmOC) remains one major challenge in addressing the burden of maternal mortality worldwide [4].

Though the introduction of the free maternal care policy in Ghana in 2008 was geared towards increasing access to maternal health services across the country, ensuring that women get the needed care on time has been a challenge. Despite global and national efforts to improve maternal health, Ghana failed to achieve the Millennium Development Goal 5 of improving maternal health by the year 2015 [5]. Delays from different angles have been seen as some of the underlying factors that contribute to high maternal mortality in LMICs. Thaddeus and Maine [6] explain these three (III) delays to include: (I) delays in recognizing danger and deciding to seek care, (II) delays in reaching the appropriate facility, and (III) delays in receiving quality care once the woman reaches the facility. Addressing the second and third delays are vital to a proper functioning referral system.

Ghana’s health system provides for three tiers of healthcare; primary, secondary, and tertiary and the referral system is used as the main link among them. The referral system requires patients to first access primary care and be referred to the appropriate level when the need arises [7]. Tertiary hospitals are the final referral points where care is given by specialists for complex cases. The function of referral is of particular importance in pregnancy and childbirth, as a range of potentially life-threatening complications require management and skills that are only available at higher levels of care [8]. In 2012, the Ministry of Health came up with a national policy to help address delays in accessing emergency care for referred patients [7]. This referral policy sought to ensure harmonisation of the referral system for better collaboration and communication between health facilities. The implementation of this policy to achieve its intended purpose has however remained problematic.

Researches on emergency obstetric referrals in Africa [9–11] and elsewhere [12] have highlighted issues such as formalised communication and transport arrangements, adequately resourced referral centres, physical space and agreed specific protocols for referrer and receiver as some of the requisites for successful maternal referral systems. However, research on emergency obstetric referrals in Ghana appear to be limited. Afari et al. [13] and Awoonor-Williams et al. [14] examined the referral of obstetric emergencies in poor resource settings in Ghana and audited maternal and new born emergency referrals in northern Ghana respectively. These studies focused on primary and secondary health facilities with no information on referrals at tertiary levels which deal with large numbers of very complicated cases. In one study, Nkyekyer [15] conducted a descriptive study of peripartum referrals to a tertiary health facility (Korle Bu Teaching Hospital). He identified inadequacies in some aspect of the referral mechanism but little information was given on communication of transfers and feedbacks, and other health system factors which affect emergency care.

This study aimed to identify barriers associated with the referral of emergency obstetric cases to tertiary health facilities in Ghana using Korle Bu Teaching Hospital (KBTH) as a case study. Examining the lived experiences of patients, healthcare providers and relatives of patients on emergency referrals to the obstetric unit at KBTH are crucial in advancing ideas for improvement and sustainability.

## Methods

### Study setting

Korle Bu Teaching Hospital was established in 1923 and located in the capital city of Ghana, Accra. It is currently the third largest hospital in Africa and the leading national referral centre in Ghana. The hospital currently has a 2000 bed capacity and 17 departments/units serving an average of 1500 patients a day. The Obstetrics and Gynaecology department serves mainly as a referral centre for the southern part of the nation, which has a population of over 10 million. The Obstetric unit of the Obstetrics and Gynaecology department has two labour wards and 275 beds for obstetrics cases. The unit receives referral cases from other lower level facilities both public and private within and outside Accra. Often, more than half of all the referred cases are of emergency nature. The unit records several pregnancy and delivery complications in a year with hypertensive diseases mostly at the top. The unit has in time past had more than 80% of referral cases resulting in maternal deaths each year [16]. A wide range of services ranging from minor through to major procedures are conducted by health professionals in the unit. The unit has two operating theatres and these theatres have a five-bed recovery ward.

### Study design

The study employed the qualitative method and used phenomenology. The social constructivist paradigm was used as a guide for the study. This paradigm acknowledges the subjective and multiple nature of reality and posits a very close contact with the participants being studied [17]. Assumptions made under this paradigm suggest that individuals develop subjective meanings of their experiences as a way of seeking understanding of the world in which they live and work. Social constructivists however state that these subjective meanings are not imprinted on individuals but are rather co-constructed with others through interaction [18]. This presents an opportunity for several individuals to share or describe their experience; an approach in qualitative inquiry referred to as phenomenology [17]. This approach was used to understand the lived experiences of providers, patients and relatives of patients on the challenges associated with accessing emergency care at the hospital. An interview guide was developed to elicit views from respondents.

### Study population, selection and recruitment of participants

The study population included all women who were referred under emergency conditions to the obstetric unit of the hospital, all relatives of emergency referred patients and all healthcare personnel in that unit. Study participants were selected using purposive and convenience sampling techniques. Key informants such as Doctors, midwives and nurses at the Obstetrics unit of the Obstetrics and Gynaecology department of the hospital were purposively selected for In-Depth Interviews (IDIs) because they have the knowledge in the management of obstetric cases in the facility. Data was collected from 34 health providers comprising 16 medical doctors, 13 nurses and 5 midwives. Again, IDIs were conducted with 31 purposively selected patients. The health staff at the unit were contacted to help in the selection of women who were referred from other facilities (public or private) under any form of emergency (those requiring immediate attention) within and outside Accra. However, participation in the study was guided by health professionals in selecting those who are fit primarily and willing to take part in the study. These included in-patients and discharged patients. Self-referred women and those who reported with no emergency issues were excluded from the study. Convenience sampling technique was used to select 24 relatives of patients for IDIs comprising 17 husbands, 4 sisters, 1 in-law and 2 mothers. Relatives of patients were contacted during visiting hours and those who agreed to take part in the study were interviewed at a time and place of their preference. In all, a total sample size of 89 was determined using a saturation approach [19].

### Data collection

Eighty-nine (89) face-to-face IDIs were conducted with the help of semi-structured interview guides which were pre-tested before their final adoption. This exercise was undertaken after an approval was granted by the Scientific and Technical Committee and Institutional Review Board of the hospital. Questions listed in the guides were open ended and had the principal aim of eliciting responses from healthcare providers, patients and their relatives on the referral of emergency cases to the hospital and the challenges associated with it. Views on how to ensure effective referral and management of emergency cases to the hospital were also sought from all participants. All IDIs were conducted by two trained nursing students. Interviews were conducted in English and two local languages (Twi and Ga). The interview guides were not translated into the local languages but the research assistants were taken through rigorous training to accurately translate the interview guides into the two local languages to ensure consistency. An arrangement was made to present the study to all healthcare providers and a date was agreed on to commence the data collection. The purpose of the study was again explained to all participants before the interviews. Participants read and signed the consent forms accordingly before each interview. For participants who could not read, the research assistants read the content of the forms after which they signed or thumb printed. Healthcare providers were visited in the wards and in their offices within the unit for the interviews. Interviews with patients were also in the wards while some relatives were also interviewed in the wards during the visiting hours. Other relatives however chose a place outside the wards for the interviews. Responses from participants were recorded using a digital voice recorder. However, responses from participants who did not consent to audio recording were handwritten. An opportunity was however given to respondents to crosscheck what was recorded after which they signed to validate the information given. The study was aided by two different sets of semi-structured interview guides (see Additional file 1); one for healthcare providers and the other for patients and their relatives. Participants were assured of confidentiality and anonymity with regards to responses given. They were also reminded that participation was voluntary and thus they could freely withdraw from the study when they deem it necessary. All interviews with participants lasted between 20 and 35 min.

### Sources of data

This study made use of both primary and secondary data. Primary data was sourced from in-depth interviews with healthcare providers, patients and their relatives. Secondary data was obtained from review of policy and guidelines on referrals and annual statistical report of the obstetric unit of KBTH. Information on obstetric emergency cases and maternal death records were sourced from the unit’s annual statistical records.

### Data management and analysis

Analysis of data involved various phases of thematic analysis [20]. All voice recordings from interviews were transcribed verbatim. Responses in the local languages (Twi and Ga) were translated into English with the help of experts prior to analysis. To ensure accuracy, recordings in Twi and Ga were compared with the English versions for accurate translations while listening to the original voice recordings. Initial ideas were given codes while reading the data. Handwritten responses were also typed and saved in Microsoft word documents. All transcripts were stored in softcopy formats in password secured folders on a password-protected laptop computer. Transcripts were entered into Microsoft excel to help in identifying common themes regarding barriers to effective referral and management of emergency obstetric cases to the teaching hospital. Codes were collated and repeated patterns identified across the data set were identified as themes using the referral policy and guidelines and the three-delay model as a guide. Four (4) global themes were developed through a review of the various themes. The next step involved the refinement of the global themes which led to the development of sub-themes. Finally, compelling extracts of the data were used to back analysis in relation to the objectives of the study.

## Results

The study identified a range of challenges encountered in the referral process and other issues which affect quality of care. Most of the major challenges identified in the transfer of obstetric emergencies from the lower facilities to the teaching hospital have influence on type II and III delays in accessing healthcare as espoused by Thaddeus and Maine [6]. These included communication barriers, referral transportation system, health infrastructure and supplies and human resource issues.

### Referral transportation system

#### Availability of ambulance services

On the issue of patient transportation, respondents unanimously agreed that the best mode of transportation was the ambulance, however for various reasons primarily the limited availability of ambulances and finances, many of the patients arrive in taxis or private cars. Some health personnel had this to say:

*“They commonly come in taxis and then sometimes ambulance and private cars….It will be preferable that they should all come in an ambulance so what we have now is not the best”*[Midwife, worked for 2 years]*.*

*“Some by ambulance and others by Taxis. ….Some of them we don’t think they should be in taxis but they come in them, a few from big hospitals bring an ambulance so those with the taxis are not the best*” [House officer, worked for 7 months].

Similar sentiments were shared by some patients and a relative:

*“We took a taxi….. I suffered before getting the taxi. There was no taxi around so they had to walk towards the police station before securing one and I was still bleeding”* [Patient referred with severe bleeding].

*“We were told to go to Korle Bu and they didn’t give us an ambulance so we took a taxi. My sister went to the back of the hospital and got the taxi so……”* [Patient referred with postdate condition]*.*

*I had to look for a taxi and send her… Since it wasn’t day break yet, it was difficult securing it”* [Sister to a patient referred with severe anaemia].

On the issue of using other forms of transport other than an ambulance, some relatives of patients emphasized the importance of using ambulances and described some of the challenges they encountered on the way with respect to accessibility and safety. Some views are expressed below:

*“…..there was heavy traffic and other cars don’t want to give way even though the driver was blowing the horn. If it was to be an ambulance, we could have reached here much earlier”* [Sister to a patient referred with severe bleeding].

*“…..as i said, the second birth, [she] had some complication. We came in an ambulance and that was much better. When they hear the ambulance, they give way but for the taxis some think maybe the taxi driver is trying to be smart so they refuse to give [them] way…”* [Husband; wife referred with placenta abruptio].

#### Poor management of patients during transit

Transporting patients during emergency transfers in ambulances help to ensure continuity of care as paramedics are able to monitor and provide necessary first aids to help stabilize patients’ conditions before they arrive at the referred facilities. Carrying patients in inappropriate transports compromise on these interventions and the safety of patients thereof. Some health professionals shared their experiences:

*“……because they normally come with taxis and private cars, the condition of patients get worsened, they don’t come in with required ambulances which […] could help stabilise them…”* [Nurse, worked for 3 years].

*“……….in the ambulance, there are life support equipment to support the patients whilst coming like oxygen administration, running intravenous transfusion and proper monitoring of patient before they arrive as compared to the taxi where these equipment can’t be placed inside to keep the patient hydrated…”* [House officer, worked for 8 months].

#### Lack of professional escort

Majority of the health professionals indicated that referred patients were often unaccompanied by health professionals. Respondents believed this was because the referring hospitals and clinics were short-staffed or their nursing staff were lazy and unwilling to accompany the patients. There were also situations where patients who arrived with ambulances at the hospital were not escorted by health professionals. The views of some health professionals are expressed as follows:

*“Not always, most time patients come with their relatives few times nurses escort them. The referring facilities do not have enough staff to escort the patients, that is what they always say and they are aware it’s not the right thing to do”* [Nursing officer, worked for 4 years].

*“Patients are escorted by nurses and sometimes they come alone with their relatives. Mostly those who come by ambulance are escorted and at times too patients are not escorted even though they come by an ambulance”* [Nursing officer, worked for 6 months].

*“…..even if [they] are not bringing the patient in an ambulance, at least they should come with a nurse because anything can happen on the road so it’s better if an assisted nurse is with her to give other information we will need if the patient can’t respond”* [House officer, worked for 6 months].

### Poor communication between referral-receiving hospitals

#### Unannounced emergency referrals

Majority of health professionals reported that ideally the referral process begins with communication from the referring hospitals and clinics through telephone calls. However, they indicated that sometimes referring facilities forgo the telephone calls. The health workers interviewed opined that this could be mainly because these hospitals may not have KBTH’s emergency number or that they want to avoid being informed that there is limited bed availability and would thus have to refer the patients elsewhere. Voices of some health professionals are shared below:

“….*Talking of those who don’t call Korle-Bu before bringing the patient, it can be like they don’t have our number or they are in a haste to bring the patient because of exacerbation of case”* [House officer, worked for 5 and a half months].

“[ ]*Those who call, call on the emergency unit phone…[ ] Others have the numbers but choose not to call because they think we might tell them the place is full so for all you know, you hear the siren blowing and an emergency is at your door step*” [Resident doctor, worked for 6 years].

#### Lack of adequate referral information

Once at the hospital, the patients or their escorts (relatives or health professionals) are expected to present their referral forms. These forms contain a summary of the patients’ personal data, a little medical history, diagnosis, and treatments given. However, some of the health professionals who were interviewed observed that though most patients come with referral letters sometimes the information provided by the referring facilities are inadequate.

*“…at times we have to call those facilities to get those information on them [*]… *it delays the care of patients. At times the information they write on the referral letters are wrong so at times we don’t base with the referral, we do our own examination and then come out with our own diagnosis…”* [Midwife, worked for 2 years].

*“….some of them maybe is the way they manage the patients. If you get to know the information, we will blame them so they will hide some of them [ ] ….but those who manage well give details”* [Medical doctor, worked for 2 years].

#### Poor feedback system

On the issue of giving feedback to referring hospitals after the patient had been treated, majority of health professionals reported that KBTH mostly did not provide referring hospitals with feedback on the patients. However, some reported that feedbacks are given upon request and sometimes informally through referral escorts. Time was one of the major reasons given for not carrying out this exercise. Some views are shared as follows:

*“..[ ]unless the referring facility calls to ask for information about their patient. There is no channel to convey that information, no one has the time to do that, there are enough and overwhelming patients to attend to than busily giving feedbacks”* [Resident doctor, worked for 6 years].

*“As for feedbacks, [ ] … we see it as not necessary because at the end of the day, it’s the patient we want to keep away from danger so if patient recovers, and she is discharged. No one has time to call or give feedback to the referring facility”* [Nurse, worked for 4 years].

However one respondent observed that there was a section of the referral that required feedback for the referring hospital but that the hospital staff often neglected this duty.

*“I don’t think we do well in that particular regard because for the referral, there is a part that you are supposed to fill to give feedback but there are times too we call back the facility and try to correct errors as in where we notice them. There is no particular reason why we don’t do it but I think it should be done”* [Midwife, worked for 5 years].

### Inadequate infrastructure and supplies

#### Limited bed availability

Regarding the availability of beds, all the health professionals agreed that the limited number of beds had a negative influence on their ability to attend to patients. A majority of respondents indicated in certain instances, the unavailability of beds forced the unit to shut down the emergency ward or refer patients to other hospital which delayed treatment and often exacerbated the situation.

“…*it short circuits the care, because if there are no beds, we either have to close down the emergency or maybe turn the patients away. If no bed, there is usually not more we can do”* [House officer, worked for 6 months].

*“Bed really affects care given so if there is no bed, no admission so if the hospital calls that they are bringing emergency cases, we just tell them the place is full. Others who came in without calling us we send them back, [ ] we give the necessary first treatment and refer them to either 37 military hospital or Ridge…[ ] We cannot nurse a patient on the floor and some special cases need special position to nurse them in bed so if there are no beds, no admission*” [Resident doctor, worked for 6 years].

#### Inadequate drug supplies and blood banking services

Most of the respondents emphasized the need for an adequate supply of equipment and other necessary medical supplies especially blood and drugs as inadequacies often bring patient care to a halt. Some health professionals explained these challenges to providing emergency care:

*“…..that’s the house officers headache [….]Sometimes, there is no blood so patients come in bleeding and they are sent away, sometimes the pharmacists are on strike and so drugs are not available so unless we send them outside the entrance of the hospital and then they have to buy especially at night, it’s very difficult …[..] …and the equipment sometimes, you need some emergency labs and it’s not available, we don’t have an emergency room that contains everything to manage such a case”* [House officer, worked for 9 months].

Many relatives shared their frustration and fears in having to go and look for drugs needed for emergency cases. These sentiments were captured in the voice of one respondent, *“[…].but if my wife is dying but I don’t have money, you have written down the medicines for me, where am I going to get the money to pay for that? So if they rather take care of her then later write out my bill for me that will be better”* [Husband; wife referred with Postpartum Hemorrhage (PPH)].

Another relative echoed*,” They take care of the patient but the situation where you will have to go and buy the medicine before they treat the patient is what worries me”. “It was the medicines that they said they don’t have at the pharmacy so we should go outside Korle-Bu teaching hospital to buy”* [Husband; wife referred with severe anaemia].

Similar frustration was shared by a patient, *“For instance, the lab had closed when we got there and the drugs we were asked to buy, when we went there, we were told it’s finished so they asked us to go to a different pharmacy…”* [Patient referred with high blood pressure].

### Insufficient health personnel

Proper attention and care given to emergency cases are mostly reliant on the availability of health personnel. The numbers involved in discharging these duties have been seen to be of significant importance especially for a tertiary facility which receives high level of referrals from several quarters. Health personnel at the emergency unit called for extra hands in giving timely care to emergencies that get there through confirmed and unannounced means. A respondent opined:

*“My problem is based on the question you asked about how we manage emergency patients. I will say the hospital is doing well but if there is chance available we need more staff because sometimes the work becomes very tedious”* [Nurse, worked for 3 years]*.*

Another personnel added, *“[...]There are not enough health personnel at the emergency because you will come to work and maybe 1 mid-wife 1 doctor taking care of maybe 3 or 4 bad cases”* [Nurse, worked for 4 years].

A relative also shared in their opinion, *“My problem is with the nurses, I think there should be more nurses at a time at the emergency side like Sunday’s or weekends. There should be more health personnel there to assist the doctors and mid-wives so that the job will be easy for them”* [Husband; wife referred with severe anaemia].

#### Patients-providers challenges on quality of care

Although not directly related to delays in receiving care at the referral centre, patients and relatives highlighted challenges they encountered at the hospital and included: addressing poor treatment given to relatives of referred patients by health professionals and improving the living conditions in the wards.

## Discussion

Findings from the study identified major challenges in the referral process for emergency obstetric cases and receiving care at the tertiary health facility. Major domains identified included: transportation system, communication barriers, infrastructure and supplies and health personnel.

Lack of appropriate transport for emergency obstetric cases were identified as a major challenge by all the respondents for the study. This however falls out on the requisites for a successful maternal referral systems as advanced by Murray and Pearson [9]. Similar findings were also noted in studies on emergency obstetric referrals in primary health facilities in Ghana [13] and Ethiopia [10]. Most of the obstetric emergencies that arrived at the teaching hospital were in taxis or private cars and confirms an earlier finding in the facility [15]. The choice of this form of transportation for emergency cases has been described as the current norm in many Low and Middle Income Countries [21]. Though the availability of public and private transport may not be a problem in urban areas, the preference for using ambulance in transporting emergency cases is underscored by safety and accessibility reasons. The use of ambulances help to ensure continuity of care during the transit period by paramedics and also guard against delays in travelling through busy cities. Non-use of ambulance service according to most of the respondents were attributed to availability. Many public and private health facilities have no or few ambulances serving several needs. National ambulances stationed at designated points are busy and not dedicated to obstetric emergencies only. This makes their availability in obstetric referrals unreliable. More than 50% of health facilities in Ghana arrange with private parties (taxi) for emergency referral [22]. Even though relatives of patients had issues with the general cost of transportation to the facility, it did not serve as a barrier to accessing care due to the emergency nature of the cases. The National Ambulance Service (NAS), instituted in 2004, sought to provide emergency care and transport to patients for free. This service is however fraught with several logistic challenges. Accra was reported to have the largest complement of ambulances and personnel: 8 ambulances and approximately 100 Emergency Medical Technicians (EMTs) [23]. The reliance on ambulance services for emergency transfers was not confirmed by this study and this is reflective of a study by Zakariah et al. [24] where obstetric conditions formed only 24% of the total patients cared for by the ambulance service in the country. Poor ambulance service as reported by some health personnel should be addressed as a similar finding has shown to affect emergency obstetric care in a teaching hospital in Nigeria [11]. Emergency transport system organised for maternal emergencies in other countries such as Sri Lanka [25] and through Public Private Partnership in India [26] could be replicated in Ghana to improve emergency referral to health facilities.

Communication is needed among other things, to arrange prompt referral and allow the receiving facility to prepare for the emergency [7, 27]. Findings from this study mostly discounted the importance of this exercise as the hospital had a number of emergency cases coming in unannounced. This challenge is emphasized by other studies [10, 13]. The advent of mobile telephones was seen as vital to enhancing communication for referrals in LMICs [28]. Several challenges were identified with phone communication between referring facility and the teaching hospital. In as much as other reasons such as fears relating to non-acceptance of referred cases due to bed unavailability were purported to account for non-prior communication on referrals, health professionals acknowledged that the communication gap could be due to phone directory challenges with some of the facilities. However, the practice of referring complicated cases to higher facilities has also been seen as an attempt to evade responsibility from fear of being blamed by patients’ families or being questioned by higher authorities in the likely event of adverse outcomes [29]. This increases the likelihood of multiple transits and subject women to further risk of death. The obstetric unit of the hospital recorded 86.6% of referrals resulting in maternal deaths between 2011 and 2015 [16]. Procedures to advance prior arrangements to receive emergency cases are vital to preventing maternal deaths. A report by the MOH et al. [22] give credence to this finding as a national challenge and states that facilities were less likely to call ahead to inform on referrals to receiving facilities. Contrary finding to this national challenge is however seen from a study in Malawi [30] in a criteria-based audit of a District Referral System for obstetric emergencies where calling ahead to inform on referred cases was the practice.

Again, the adequate provision of referral information is important to ensuring timely continuity of care at referred centres. Patients referred to the unit were accompanied by referral slips in line with the national referral policy and guidelines and same findings were observed in other LMICs [11, 30, 31]. Health professionals however recounted that comprehensive information is not always given which informs re-diagnosis and calling referring facilities to provide further details on care given. Going through such an exercise becomes necessary at a time but however delays care given to an emergency case. Chaturvedi et al. report on similar finding in a quality assessment of obstetric referral services in India [31]. Providing formal feedbacks to referring facilities were mostly ignored in this study and this confirms results from Omo-Aghoja et al. study in a teaching hospital in Nigeria [11]. Giving feedback to referring facilities is not only a problem with teaching hospitals but other levels of healthcare as revealed by Awoonor-Williams et al. [14] in northern Ghana. The governing structures of the various health facilities: teaching hospitals, public facilities, private facilities and faith based facilities have implications on regulation and coordination with respect to referrals and healthcare delivery. Teaching hospitals are autonomous and are thus outside the direct control of Ghana Health Service. Ensuring an effective referral system will thus need a strong collaboration and coordination among the various levels of care. Appropriate measures should be put in place to address communication barriers so as to help address some of the problems identified in the transfer of obstetric emergencies.

There is also the need to adequately resource referred centres so as to meet the various needs for obstetric emergencies. Consistent with our findings, several studies in Africa [11, 32, 33] and elsewhere [12] have underscored the need for such provisions to improve maternal health. Issues with bed availability, blood banking services, drugs and personnel identified in this study contribute to type III delays as indicated by Thaddeus and Maine [6]. Deciding to seek care may be necessary but not sufficient to avert adverse outcomes if the process to receive prompt care is delayed at the health facility as alluded to by one of the authors in a maternal death audit in rural Gambia [33].

### Study strengths and limitations

This study provides information on the referral of obstetric emergencies to a tertiary health facility in Ghana. It brings to the fore an understanding of the inherent challenges in the system using multiple stakeholder perspectives. The study contributes to the body of knowledge of obstetric emergency referrals to tertiary hospitals, in a country where there is little research conducted on the referral process and other challenges encountered in receiving care at tertiary health facilities.

While study results may not be generalizable to other tertiary health facilities, they can offer insights and useful recommendations which could lead to improved referral system for obstetric emergencies when applied. Additionally, the study only focused on the receiver end of the referral system and to have a comprehensive view of the challenges of the entire system it will be prudent to involve sending facilities. Similar studies have to be conducted in referring facilities to help inform appropriate interventions needed to improve the entire referral system for obstetric emergencies.

## Conclusions

High maternal deaths recorded in many developing countries could be averted by effective referral systems. Initiatives to improve the transportation system for obstetric emergencies are vital in ensuring patients’ safety and continuity of care during transfer. Communication between referring and receiving facilities should be enhanced. A strong collaboration and engagement between teaching hospitals and public facilities, Christian Health Association of Ghana and private facilities is needed to boost communication on referrals and healthcare delivery. Concurrently, supply side barriers at referred facilities must be addressed. These include ensuring sufficient provision for bed, blood, drugs, equipment, personnel and creating a conducive environment for other stakeholders such as patients’ relatives.

## Supplementary information

**Additional file 1.** Interview guides.

## Data Availability

The datasets used and/or analysed during the current study are available from the corresponding author on reasonable request.

## Abbreviations

AMDD: Averting Maternal Deaths and Disability
CHAG: Christian Health Association of Ghana
EmOC: Emergency Obstetric Care
EMTs: Emergency Medical Technicians
GHS: Ghana Health Service
IDIs: In-Depth Interviews
KBTH: Korle Bu Teaching Hospital
LMICs: Low and Middle Income Countries
MOH: Ministry of Health
NAS: National Ambulance Service
NDPC: National Development Planning Commission
SDGs: Sustainable Development Goals
UN: United Nations
UNFPA: United Nations Population Fund
UNICEF: United Nations Children’s Fund
WHO: World Health Organisation
