# Supplementary material for: Improving emergency obstetric referral systems in low and middle income countries: a qualitative study in a tertiary health facility in Ghana
Source: BMC Health Serv Res. 2020 Jan 10;20:32. doi: 10.1186/s12913-020-4886-3 (PMC6954606; doi:10.1186/s12913-020-4886-3)
Supplement: Supplementary file 1 — Additional file 1. Interview guides. [file 12913_2020_4886_MOESM1_ESM.pdf]

INTERVIEW GUIDE FOR HEALTHCARE PERSONNEL

Dear respondent, thank you very much for agreeing to do this interview with me today. Studies have shown that many maternal deaths results from complications during and following pregnancy and childbirth and timely management and treatment can make the difference between life and death for both the mother and the baby. It is against this background that we seek to know your opinions about the referrals of emergency obstetric cases to this facility.

This is purely an academic exercise and as such all responses will be treated with the strictest confidence.

Time Interview began……………………………………

Item	Section 1- Background Information	Response
1	Sex	
2	Position of respondent	
3	Interview date	
4	Interviewer Name	
	**Section 2- Assessment of the referral process**	
5	How long have you worked as a health facility staff in this unit?	
6	What type of Obstetric conditions are usually referred to this unit?<br>**Probe:**	
	1. How many emergency referred cases do you receive in a day?	
	2. Type of facilities referred from and their location	
7	What is normally the means of transportation for referred emergency cases to the unit?	
8	What is your opinion about the means of transportation of emergency cases to the facility?	
9	Are patients always escorted during referrals?<br>If yes, which people escort them?<br>If No, what do you think account for that?	

10	Do referring facilities always communicate with KBTH before sending emergency cases?<br>If yes, what is the mode of communication, and what time is this done in the transfer process?<br>If No, what accounts for this, and how does the unit handle such cases when they come?	
	**Probe:**<br>1. Is there a call center or a person designated to attend to calls on referrals to the unit?	
	2. If Yes, who receives this call?<br>If No, how do other hospitals contact this unit?	
11	If forms are used: Do incoming patients bring referral forms always, often, sometimes or never?	
	**Probe:**<br>Do all patients referred to this unit bring a standard referral form/ the same referral form?	
12	Please describe what information patients referred to this unit usually bring.<br>**Probe:**	
	1. Would you say that referring facilities provide enough information about the patients and details of treatments given already?<br>If No, what do you think account for that and how does that affect the management of the patients.	
13	How does this unit provide feedback to referring facilities after patients have received care?<br>**Probe:**	
	1. What information is given and how important and frequent is this exercise?	
	2. If feedbacks are not mostly given, what accounts for that?	

14	What is the procedure for receiving emergency cases referred to this unit?	
15	How do issues of bed availability affect the care given to emergency referred cases to this unit?	
16	How do issues of cost affect the prompt delivery of care to referred cases to this unit?	
17	How do availability of equipment, blood and supplies affect the prompt delivery of care to emergency referrals to this unit?	
18	How does the availability of health personnel influence prompt delivery of care to emergency cases brought into this unit?	
	**Probe:**<br>In your view, do you think that there are enough health personnel available at any point in time to attend to emergency cases?<br>What do you have to say about your answer please?	
19	Do you have any recommendations on how emergency referrals could be improved in this unit?<br>If so, could you please tell me?	
	**Probe**: Do you have other suggestions?	
22	Do you have any other comments that you would like to make with regards to emergency referrals to this unit which hasn’t been addressed?	

Time Interview ended……………………………………..

Thank you very much for your time and .cooperation

IN-DEPTH INTERVIEW GUIDE FOR PATIENTS/RELATIVES OF PATIENTS

Interviewers are to note that patients who are declared as medically fit to take part in the study by health professionals are those who can be considered for interviews.

Time interview began………………………………

Item	Background Information	Response
1	Health Insurance Status of patient.	
2	Condition of referral / Relationship to patient	
	**Assessment of the referral process**	
3	Were you told why you had to be referred to Korle Bu, the care you were to receive here and the cost involved?	
4	How was the decision to come to Korle Bu made and who were involved in the decision making?	
5	Which hospital were you referred from and where is it located?	
6	Which transport service did you come with to KBTH and how did you secure it?	
	**Probe:**<br>How easy or difficult was it to secure this form of transport?	
7	Who paid for the cost of transportation and how affordable was the cost involved?	
8	How long (in terms of time) did it take you from your referral facility to get to KBTH?	
	**Probe:**<br>1. Do you think that you could have travelled to this unit in a lesser time than stated above?	
	2. If yes, what opinion/situations or choices could have saved the situation?	
9	How early/long were you attended to by the health professionals when you arrived at the unit?<br>**Probe:**	

	1. Were you satisfied with the speed or attention given to you by the health professionals when you arrived here? Can you please share your experience with me?	
10	Did you make any payment when you got to this facility? If yes, at what point or time were you asked to make payment at this unit?	
11	What specific items/ services were you made to pay for?<br>What would you say about the payments you were made to pay in terms of affordability?	
12	How would you describe the general attitude of health personnel with regards to emergency cases referred to this unit?	
13	In your view, do you think that there are enough health professionals available at any point in time to attend to emergency cases	
14	What would you say about the availability of needed materials to attend to emergency cases?	
	**Probe:**<br>1. Did you experience anything about the non-availability of certain materials or items where you had to wait for some time or find a way to procure it yourself when you got here?	
	**2.** What specific material or item was involved in your case?	
15	Do you have any other issue/ experience you want to share with me with regards to your referral to this unit?	
16	Do you have any recommendations/ suggestions on how emergency referrals to this unit could be improved? If so, could you please tell me?	

Time interview ended……………………………………………

Thank you very much for your time and cooperation.
